# Is oropharyngoesophageal scintigraphy the method of choice for assessing dysphagia in systemic sclerosis? A single center experience

**DOI:** 10.1007/s10388-023-00995-0

**Published:** 2023-03-18

**Authors:** Marco Di Battista, Mariano Grosso, Mattia Da Rio, Giammarco De Mattia, Andrea Marciano, Anastasiya Valevich, Antonio Grosso, Riccardo Morganti, Duccio Volterrani, Alessandra Della Rossa, Marta Mosca

**Affiliations:** 1 Rheumatology Unit, University of Pisa, Via Roma 67, 56123 Pisa, Italy; 2 Department of Medical Biotechnologies, University of Siena, Siena, Italy; 3 Nuclear Medicine, Department of Translational Research and New Technology in Medicine, University of Pisa, Pisa, Italy; 4 Department of Translational Sciences and New Technologies in Medicine and Surgery, Gastrointestinal Unit, University of Pisa, Pisa, Italy; 5 Section of Statistics, University of Pisa, Pisa, Italy

**Keywords:** Systemic sclerosis, Dysphagia, Oropharyngoesophageal scintigraphy, Barium esophagogram

## Abstract

**Objectives:**

To evaluate the performance of oropharyngoesophageal scintigraphy (OPES) in the assessment of dysphagia in patients with systemic sclerosis (SSc), and to compare OPES results with those of barium esophagogram.

**Methods:**

Adult SSc patients who underwent OPES for the assessment of dysphagia were enrolled. OPES was performed with both liquid and semisolid boluses and provided information regarding oropharyngeal transit time, esophageal transit time (ETT), oropharyngeal retention index (OPRI), esophageal retention index (ERI), and site of bolus retention. Barium esophagogram results were also collected.

**Results:**

Fifty-seven SSc patients (87.7% female, mean age 57.7 years) with dysphagia were enrolled. OPES identified at least one alteration in each patient and findings were generally worse for the semisolid bolus. Esophageal motility was widely impaired with 89.5% of patients with an increased semisolid ERI, and middle-lower esophagus was the most frequent site of bolus retention. However, oropharyngeal impairment was highlighted by widespread increased OPRI, especially in anti-topoisomerase I positivity. Older patients and with longer disease duration presented slower semisolid ETT (*p* = 0.029 and *p* = 0.002, respectively). Eleven patients with dysphagia had a negative barium esophagogram: all of them presented some alterations in OPES parameters.

**Conclusion:**

OPES revealed a marked SSc esophageal impairment, in terms of both slowed transit time and increased bolus retention, but also shed light on oropharyngeal swallowing alterations. OPES showed high sensitivity, being able to detect swallowing alterations in dysphagic patients with negative barium esophagogram. Therefore, the use of OPES for the assessment of SSc-related dysphagia in clinical practice should be promoted.

## Introduction

Systemic sclerosis (SSc) is a chronic connective tissue disease which is pathogenically driven by microangiopathy and autoimmune dysregulation, both leading to fibrosis of skin and internal organs. From a clinical point of view, SSc is a very heterogeneous condition and gastrointestinal involvement can be found in up to 90% of patients. Esophageal dysmotility is very frequent, and can result in burdensome symptoms such as gastroesophageal reflux and dysphagia, thus determining an increase of morbidity and a worsening of the quality of life [1, 2].

Several diagnostic techniques to investigate esophageal dysmotility have been proposed so far, including barium esophagography and manometry. Among them, oropharyngoesophageal scintigraphy (OPES) emerged as an easy, cheap, and well-tolerated non-invasive method to assess dysphagia, as it allows both a functional and a semi-quantitative study for each stage of swallowing, thus representing a useful technique for all the different kinds of dysphagia [3–5].

The aims of this study were to evaluate the performance of OPES in the assessment of dysphagia in SSc patients looking also for possible associations with disease characteristics, and to compare OPES results with those of barium esophagogram.

## Methods

### Patients

Adult patients routinely followed at the Rheumatology Unit of the University of Pisa and fulfilling 2013 EULAR/ACR classification criteria for SSc [6] who underwent OPES per routine clinical practice for the assessment of dysphagia, were included in this cross-sectional observational study. Each patient voluntarily agreed to participate and gave written informed consent for the publication of the present study which was conducted following the principles of the Declaration of Helsinki.

Dysphagia was reported as one of the major complaints in each subject’s clinical history. Epidemiological and SSc-specific data were collected, including disease duration, skin subset, autoantibody profile (anti-centromere, anti-topoisomerase-I or other autoantibodies), and other major involvements (interstitial lung disease—ILD, pulmonary arterial hypertension or digital ulcers).

If performed within the previous year, data regarding the presence of esophageal dysmotility and hiatal hernia assessed by barium esophagogram were collected.

### Oropharyngoesophageal scintigraphy

OPES provides a rapid sequence of images of voluntary swallowing acts performed by the patient when asked to. Fasting at least 3 h prior to the examination was required. To get used to the procedure, each subject first swallowed about 10 mL of non-radioactive water. The investigation then started with the patient in standing position and with the face in an 80° oblique projection in front of a large-field-of-view γ-camera equipped with a low-energy parallel hole collimator and an energy window on 140 keV (± 10%). The patient was then given a single 10 mL bolus of water labeled with 37 MBq of ^99m^Tc nanocolloid (Curium, UK). Eight images per second were acquired (0.125 s/frame) for 1 min, with dynamic acquisitions (64 × 64 matrix and zoom 1) of the visual fields from the oral cavity down to the epigastric area. Two seconds after the beginning of the procedure, the patient was asked to swallow the liquid in one gulp. With the subject remaining in the same position, a 60 s lasting static image was acquired at the end of the examination, to evaluate the presence of a possible tracheobronchial aspirate. At the end of the procedure, the possible presence of multiple swallows was assessed, as these would not allow an accurate semi-quantitative analysis; in such cases, the test was repeated after having the patient drink several sips of unmarked water to wash out any residual radioactive substance left in the oral cavity. The examination was then repeated after an interval of 30 min, with a 10 mL “jelly drink” semisolid bolus (Resource Bevanda Gelificata, Novartis S.A.^®^) labeled with 37 MBq of ^99m^Tc nanocolloid. Scintigraphic acquisitions were then performed in the same way as the liquid bolus.

**Fig. 1:**
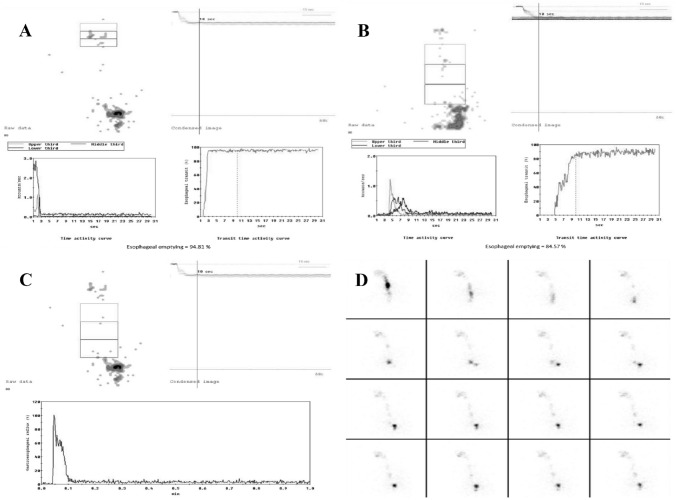
Typical time–activity curves obtained from OPES of a healthy subject (derived from [4, 7]). Normal time–activity curves for (**A**) mouth and pharynx, **B** upper third, middle third, and lower third, and **C** whole esophagus. **D** Review of the dynamic recording in cine-mode shows a regular transit bolus.

As previously mentioned, OPES allows both qualitative (cine-mode) and semi-quantitative analysis. Cine-mode visual analysis of the images acquired during bolus swallowing allows the identification of abnormal patterns such as multiple swallows, bolus retention in the oral or pharyngeal cavity, bolus fragmentation, gastroesophageal reflux, tracheal aspiration, and motility abnormalities. Semi-quantitative analysis involves the calculation of the oropharyngoesophageal transit time of the radioactive bolus, with manual definition of the regions of interest (ROI) for oral cavity, pharynx, and esophagus. The semi-quantitative parameters are derived from the activity/time curves obtained on the marked ROIs. The use of cumulative images enables better positioning of the ROIs (Fig. 1). As already described [4, 7], the qualitative and semi-quantitative parameters assessed by OPES for both liquid and semisolid bolus are:

–Oropharyngeal transit time (OPTT): It is the time required for the bolus to leave the oral cavity and the pharynx from the beginning of swallowing. OPTT normally occurs within 1.2 s.
–Esophageal transit time (ETT): It is the interval between bolus entry and its exit through the esophagus. ETT normally occurs within 10 s.
–Retention index: The residual radioactive bolus fraction in each tract 10 s after swallowing. In normal subjects, the oropharyngeal retention index (OPRI) is usually < 5%, and the esophageal retention index (ERI) < 20%. Based on this, a retention score was developed. For the oropharyngeal region, score 1 was considered for OPRI between 5 and 20%, and score 2 for OPRI > 20%. For the esophageal region, score 1 was considered for ERI between 20 and 40%, score 2 for ERI between 40 and 60%, and score 3 for ERI > 60%.
–The site of bolus retention, discriminating between the upper third and the middle-lower thirds of the esophagus.

### Barium esophagogram

The examination was performed with the patient observing an overnight fasting. A volume of 135 mL of low-density barium sulfate suspension (2.8 g/cm^3^) was administered orally within 15–20 s. The esophageal phase was evaluated by having the patient ingest barium in an upright position with dynamic recording in frontal projection. In the prone position, the patient then swallowed barium boluses to evaluate esophageal motility. Esophageal dysmotility is defined when abnormal peristalsis is assessed on at least 2 of 5 separate swallows. The patient then swallowed barium to optimally distend the esophagus to evaluate the presence of hiatal hernia, rings, or strictures that may not be visible on double contrast radiographs.

### Statistical analysis

Categorical and continuous data were described by frequency and proportion, and by mean and standard deviation, respectively. One-way ANOVA and independent-samples *t *tests (two-tailed) were applied to compare categorical data with continuous data; in addition, correlation coefficients were estimated by Pearson's correlation coefficient with the result of the significance test. Significance was set at 0.05 and all analyses were carried out with SPSS version 28 (*IBM Corp., Armonk, N.Y., USA*).

## Results

From July 2015 to December 2021, out of 350 SSc patients followed at our Unit, 57 (87.7% female, mean age 57.7 years) underwent OPES for the assessment of dysphagia; their epidemiological and SSc-specific characteristics are reported in Table 1.

**Table 1:** Epidemiological and SSc-specific data of the cohort (*n* = 57)

Female (%)	50 (87.7)
Mean age (± SD) years	57.7 (13.7)
Mean disease duration (± SD) years	5.8 (5.8)
Skin subset	
lcSSc (%)	47 (82.5)
dcSSc (%)	10 (17.5)
Autoantibody profile	
Anti-centromere (%)	29 (50.9)
Anti-topoisomerase-I (%)	10 (17.5)
Other (%)	18 (31.6)
ILD (%)	12 (21.1)
Pulmonary arterial hypertension (%)	3 (5.3)
Digital ulcers (%)	21 (36.8)

*SSc* systemic sclerosis, *SD* standard deviations, *lcSSc* limited cutaneous SSc, *dcSSc* diffuse cutaneous SSc, *ILD* interstitial lung disease

**Fig. 2:**
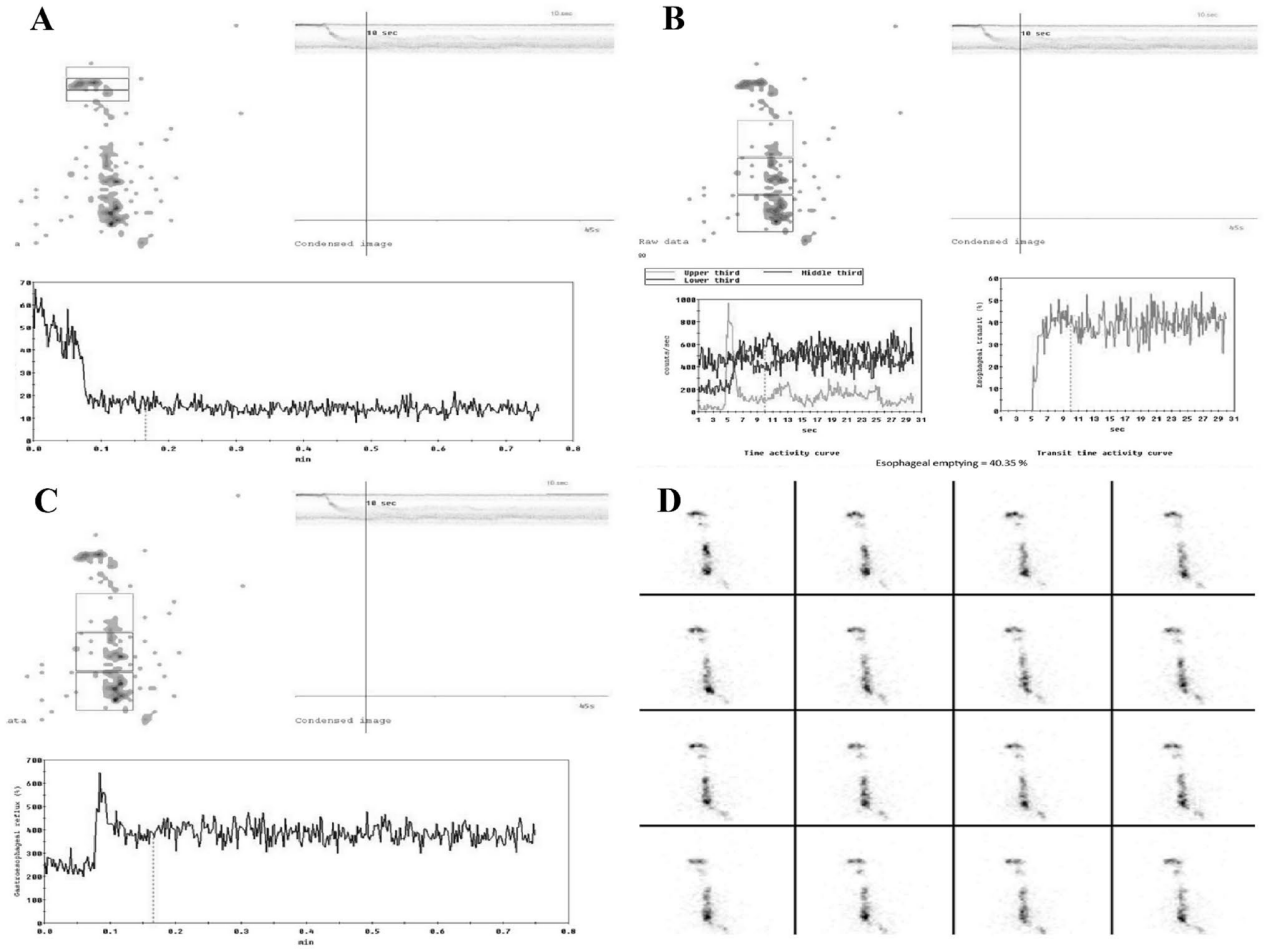
OPES with semisolid bolus of a patient with systemic sclerosis. Time–activity curves for (**A**) mouth and pharynx with marked retention of radioactivity (27%; normal values  < 5%), and for (**B**) upper third, middle third, lower third, and (**C**) whole esophagus with a marked retention of radioactivity in middle-lower esophagus (60%; normal values  < 20%). **D** Review of the dynamic recordings in cine-mode confirmed a pathologic retention in the middle-lower esophagus.

OPES identified at least one alteration in each patient (Fig. 2). Findings were worse more frequently when using the semisolid bolus than the liquid one, as reported in Table 2. Esophageal motility was impaired in terms of both transit time and retention index in approximately 30% and 60% of cases, respectively. All liquid retentions and most semisolid retentions were located in the middle-lower esophagus. Remarkably, 89.5% of patients had an increased semisolid ERI, and 9 of them had an ERI > 60%. However, OPES alterations were also found in the oropharyngeal region. Of note, most liquid OPRI and all semisolid OPRI were increased.

**Table 2:** Pathological findings at oropharyngoesophageal scintigraphy

SSc patients (*n* = 57)	Liquid bolus	Semisolid bolus
OPTT (%)	2 (3.5)	6 (10.5)
ETT (%)	8 (14)	17 (29.8)
Mean OPRI (± SD)	9.6 (5.6)	20.6 (22)
OPRI score		
Score 0 (%)	16 (28)	0 (0)
Score 1 (%)	38 (66.7)	36 (63.2)
Score 2 (%)	3 (5.3)	21 (36.8)
Mean ERI (± SD)	20.4 (11.3)	41.8 (20)
ERI score		
Score 0 (%)	39 (68.4)	6 (10.5)
Score 1 (%)	7 (12.3)	21 (36.8)
Score 2 (%)	6 (10.5)	21 (36.8)
Score 3 (%)	5 (8.8)	9 (15.8)
Upper esophageal retention	0 (0)	11 (19.3)
Middle-lower esophageal retention	17 (29.8)	35 (61.4)

When considering epidemiological data, age was directly correlated with liquid ERI (*p* = 0.009, *r* = 0.345) and mean liquid ERI score (*p* = 0.008, *r* = 0.353). Older patients had higher frequencies of slow semisolid ETT (*p* = 0.029) and middle-lower esophageal liquid retention (*p* = 0.05). Male patients had a globally higher OPRI (*p* = 0.027 for liquid and *p* = 0.048 for semisolid) and an increased semisolid ERI (*p* = 0.015) and mean semisolid ERI score (*p* = 0.023).

Evaluating possible associations with disease characteristics, patients with a longer disease duration more frequently showed a slower semisolid ETT (*p* = 0.002). Moreover, disease duration was directly correlated with semisolid ERI (*p* = 0.05, *r* = 0.261) and mean semisolid ERI score (*p* = 0.035, *r *= 0.280). No significant differences emerged for skin subsets and major organ involvement. Patients with anti-topoisomerase-I positivity had higher liquid OPRI (*p* = 0.019) and mean liquid OPRI score (*p* = 0.002).

Data regarding barium esophagogram were available for 41 out of 57 patients. This technique reported 30 cases of esophageal dysmotility and 36 of hiatal hernia. Esophagogram dysmotility was significantly associated with an increased liquid ERI (*p* = 0.008) and mean liquid ERI score (*p* = 0.036). In this subgroup of 41 patients, 11 out of 12 with middle-lower esophageal liquid retention had esophagogram dysmotility. No significant differences emerged between OPES findings and the presence of hiatal hernia.

When considering the 11 SSc patients with negative barium esophagogram (81.8% female, mean age 51.8 ± 13.3 years, mean disease duration 4.5 ± 3.9 years), several OPES parameters were pathological: one subject had a slowed semisolid OPTT, and two had a slowed semisolid ETT. Moreover, this subgroup showed an increased mean liquid OPRI (9.8 ± 6) with 6 patients reporting a liquid OPRI-score ≥ 1. Increased mean percentages were found also for semisolid OPRI (17.8% ± 7) and semisolid ERI (39.7% ± 12). Of note, all these 11 patients had a pathological semisolid score, both for the oropharyngeal region (7 patients with OPRI score 1 and 4 with OPRI score 2) and for the esophageal one (6 patients with ERI score 1, 4 with ERI score 2 and 1 with ERI -score 3). Finally, middle-lower esophageal retention was detected in one patient for liquid bolus and in eight patients for semisolid bolus.

## Discussion

OPES is as a useful tool in the assessment of dysphagia: easy-to-use, cheap, and well-tolerated by patients, it allows a non-invasive functional and semi-quantitative study of all the stages of swallowing. Determining a low dosimetric exposure (1.4 mSv), OPES can be useful and convenient both for the diagnosis and the follow-up of dysphagia [7]. In this study, we evaluated OPES findings in a cohort of SSc patients who underwent this investigation for the presence of dysphagia, which is one of the most insidious symptoms in SSc. We aimed to describe OPES alterations and, where available, we compared OPES results with those of barium esophagogram, demonstrating that OPES detects alterations in dysphagic patients even when barium esophagogram is negative.

In our study, OPES revealed a marked SSc esophageal impairment, in terms of both slowed transit time and increased bolus retention. Moreover, older age and male sex were associated with worse results: this is in line with an expected more severe disease according to the renowned epidemiological SSc features. However, we also found oropharyngeal swallowing alterations, being upper dysphagia a possible feature of SSc gastrointestinal involvement, as highlighted by Galli et al. [8]. In this context, OPES may shed light on SSc oropharyngeal swallowing impairment, which is less known but potentially frequent.

Several studies have evaluated esophageal scintigraphy in SSc; unlike these, we did not observe significant associations with diffuse skin subset or ILD [9, 10]. We found a direct correlation between ETT and disease duration as already pointed out by Åkesson et al. [11], and also an association between oropharyngeal liquid dysphagia and anti-topoisomerase-I positivity that requires further researches.

Several studies compared esophageal scintigraphy with manometry and barium esophagogram, demonstrating that the former is a valid technique with high sensitivity and specificity, and with the advantages of non-invasiveness and low dosimetric exposure [11, 12]. Our study confirms the high sensitivity of OPES, as it is able to detect swallowing alterations in dysphagic patients with negative barium esophagogram. It is worth to note that this subgroup was younger and with lower disease duration, strengthening the fact that OPES can be helpful in the early stages of SSc [13].

Our study has some limitations, mainly the retrospective design and the small number of the cohort. Furthermore, only patients who clinically complained of dysphagia were enrolled. To overcome these limitations, the use of OPES in clinical practice should be promoted, also in consideration of its aforementioned utility and clinical convenience. Other authors previously recommended esophageal scintigraphy in the routine assessment of SSc patients [14]. Our results encourage the use of OPES in the evaluation of SSc dysphagia, since OPES allows a more complete assessment of dysphagia compared to esophageal scintigraphy as it investigates also the less known oropharyngeal impairment. Another possible limitation concerns the comparability between the different boluses. The barium used in esophagogram in clinical practice has a standard viscosity, whereas boluses in OPES are made of different physiological consistencies. Moreover, it has to be said that a complete swallowing evaluation including also videofluoroscopy and manometry would have provided greater results, but this was not feasible considering that this is a retrospective study conducted in a rheumatological clinical setting.

In conclusion, given the high sensitivity and the ability to analyze the whole swallowing process, a further conceivable step could be to employ OPES in the earliest stages of the disease even in clinically non-dysphagic patients to possibly detect early subclinical involvement. However, further studies are needed for this purpose.

## Declarations

### Human and animal participants

All procedures followed were in accordance with the ethical standards of the responsible committee on human experimentation (institutional and national) and with the Helsinki Declaration of 1964 and later versions.
